# An Experiment in Regard to Cancer

**Published:** 1902-07-19

**Authors:** 


### AN EXPERIMENT IN REGARD TO CANCER.

As the result of some observations made some time ago by Dr. Bulloch, and reported by him to the Pathological Society, in regard to the transmission of hæmolysins from parent to offspring, a research of a very curious character is being made in regard to the possibility of producing a sort of antitoxic milk which shall be anti to cancer, or more accurately destructive to the cancerous protoplasm. The argument is somewhat complex, but it may be pointed out that in Dr. Bulloch's researches he discovered that by injecting the blood of the ox into rabbits the serum of the rabbits acquired a hæmolytic power towards the corpuscles of the ox, and that although this property was not transmitted by the male to its offspring by an ordinary female, the offspring of hæmolytic females gave birth to young whose serum was also hæmolytic. Incidentally, also, it was shown that young animals sucking milk from a mother immunised against tetanus acquired a very considerable immunity against that disease. This suggested the idea which is now being followed up by others, namely, that it might be possible to produce a cytolytic milk for cancer. The intention then is to disintegrate cancer of the breast and inject the expressed juice containing the broken down cell contents, into the vein of a cow, and then feed patients suffering from inoperable cancer of the breast with the milk from this cow.

